# Cerebral Surgery

**Published:** 1905-01-14

**Authors:** 


### PROGRESS IN MEDICINE AND SURGERY.

### CEREBRAL SURGERY.

**Injuries to the Spinal Cord and Spinal Column.**—Tubby[1] after describing the various traumatic affections of the vertebral column and dividing the symptoms of spinal cord injury into groups according to their position in the cord and their time of onset, gives some simple rules for treatment of these injuries. He advises immediate laminectomy in the following conditions: pressure from fractured laminæ or from a process driven inwards or a spicule of bone perforating the theca or cord, hæmorrhage, and when the cauda equina and lumbo-sacral plexus of nerves are implicated. The records up to 1901 show 37 cases recorded of immediate operation for spinal column injury; of these six recovered with extremely good results. The value of weight extension applied to the head and feet whilst the body is kept horizontal, or to the feet or head only whilst the body is placed on an inclined plane is pointed out as the best means of alleviating pain in some cases of fracture-dislocation of the spine.

**Tumours Involving the Optic Chiasma.**—Kiliani[2] describes a case of a boy, aged 16, in whom a tumour of the pituitary body caused symptoms of 14 months' duration. These were diminution of vision with bi-temporal hemianopsia, paroxysmal headache, vomiting, and exaggerated reflexes. The fundi showed hyperæmia with varicose veins. Lastly, a sudden rise of temperature to 104° Fahr., complete coma and paralysis of all four limbs occurred; an operation was performed to relieve pressure in the lateral ventricle, and the boy died. Autopsy showed a cystic adenoma of the pituitary body, measuring 3 c.m. by 4 c.m., into which recent hæmorrhage had occurred, destruction of the posterior parts of the optic chiasma, and great dilatation of the basal veins. He proceeds to describe an experimental method of reaching such tumours. A large omega-shaped osteoplastic flap, 7 inches wide and 5 inches deep, is turned up from the frontal region, the longitudinal sinus tied, the falx cut, and the frontal lobes turned back until the optic chiasma is seen.

**Fracture of Spine Complicated by Large Prostatic Calculus.**—Barton Hopkins[3] relates the case of a man of 28, who in the delirium of pneumonia jumped out of the window and fractured his spine in the region of the last dorsal vertebra. This was followed by the symptoms of complete destruction of the cord. Six months later hæmaturia and pyuria were present. After five years' suffering he, having refused all operations, shot himself. The autopsy showed, besides complete destruction of the cord just above the lumbar enlargement, and the injury to the brain caused by the bullet, a large soft calculus 6 c.m. in diameter, which occupied the prostatic urethra, and had caused septic cystitis and large double pyelonephrosis.

**Infantile Paralysis cured by Nerve Grafting.**—Harris and Low[4] showed a case of an infant, aged two years, who had had typical infantile paralysis of the right deltoid, supra-spinatus and infra-spinatus at the age of 20 months. After ineffectual electrical treatment extended over four months, an operation was performed on the brachial plexus. The fifth cervical nerve was carefully tested at the time of the operation and the upper part cut through, the distal portion of the cut nerve was then stitched into a nick made in the sixth cervical nerve. Fifteen months after the operation, there had been considerable recovery, the child being able to raise her arm and hold it straight out.

**Removal of a Cervical Intradural Tumour.**—Cushing[5] gives details of a case diagnosed by Osler and operated on by himself. A man, aged 30, for one year had had some pain in the left arm. This was followed by pain and stiffness of the neck and wasting of the muscles of the left arm, and weakness of both legs, especially the left. The left pupil was contracted. Flexion of the neck was strongly resisted. There was no loss of tactile sensation, but over the whole of the right side of the body and leg below the second dorsal segment, thermal and painful sensations were lost. About a month later loss of thermal and pain sense occurred on the post-axial side of the right arm. The reflexes were all exaggerated. The cervical spine was exposed by a median posterior incision, and the laminæ of the fifth, sixth, seventh cervical, and first dorsal vertebræ removed. An oval growth, 4 c.m. long, lay beneath the arachnoid on the left side of the cord. It was easily removed in two portions, after cutting through one of the posterior nerve roots, probably the seventh cervical. The patient made a rapid recovery. The loss of motor power on the left side and of thermal and painful sensation on the right were rapidly recovered from, the latter returning the day after the operation, and the former within a few more days. There was some loss of painful and thermal sensation in the part of the left arm supplied by the seventh nerve, but no loss of tactile sensation. Pain in the left arm and neck were never felt after the operation. References to nine other similar successful cases with brief notes are appended.

**Bullet Extracted from the Brain Ten Weeks after Injury.**—A man, aged 23, was shot in the left frontal region by a pistol bullet of low velocity. After momentary unconsciousness he walked to the train which conveyed him to the hospital. At first there were no symptoms, but three hours later vomiting, semi-consciousness and some right-sided paresis with motor aphasia occurred. After a rise of temperature to 102° F. Sheen[6] opened and cleaned the entrance wound, but no attempt was made to remove the bullet because it was so deep. Motor aphasia and right sided partial paralysis persisted for some time and paroxysms of left parietal and frontal headache made the patient anxious to have the bullet extracted. Two skiagrams from the front and the side showed the bullet to be situated 12 c.m. from the point of entrance and 6·5 c.m. from the surface of the skull above and behind the left ear. This accurate localisation enabled the bullet to be removed through an opening 3·5 c.m. above and 2·5 c.m. behind the left meatus. The patient made an uneventful recovery. Sheen suggests that the initial absence of symptoms was due to the low velocity of the bullet.

**Extensive Fracture-dislocation of the Cervical Spine producing no Symptoms.**—A boy aged 14 struck his head when diving and was taken to the hospital. Ringrose,[7] who saw him, then found no symptoms except slight transient concussion with bruising and stiffness of the neck. Ten years later, after leading an active life and having joined the Militia, the same surgeon came across him and persuaded him to have a skiagraph taken because of some slight stiffness and deformity of the neck. The skiagram showed anterior dislocation of the fifth on the sixth cervical vertebra, so that the centre of the body of the former lay over the anterior superior edge of the latter, and the adjacent vertebræ had become adjusted by compensatory curves.

**A Classification of Neuralgias by their Reaction to Cocaine Injections.**—Pitres[8] points out that if neuralgia is relieved by an injection of cocaine into the painful spot it has a peripheral origin. If the cocaine has to be injected into the course of the nerve above the painful area before relief is given, then the neuralgia is funicular. If intra-arachnoidean injection is necessary (*e.g.* in the lightning pains of Tabes) then the neuralgia is of radicular ganglionic origin. Verger similarly divides facial neuralgias into those of peripheral origin in which the paroxysm is prevented by cocaine injected into the painful area and those of more central nature not so relieved.

**Perforating Wounds of Nerve-trunks.**—Freyer,[9] as the result of his experience in the Boer War, points out that injuries to nerve-trunks are among the commonest results of high velocity bullet wounds of the limbs. But that the nerve-trunk in most instances is penetrated by the bullet, even though the latter be of much greater diameter than the nerve. The wounds generally heal rapidly by first intention, and the resulting partial paralysis eventually disappears, although some years may be occupied in complete recovery. He therefore advises non-interference with such cases, unless there is concomitant serious bone injury.

[1] Clin. Jour., Oct. 5, 1904. [2] Ann. of Surg., July, 1904. [3] Philadelphia Acad. of Surg., July, 1904. [4] Lancet, Oct. 22, 1904. [5] Ann. of Surg., June, 1904. [6] Lancet, Sept 17, 1904. [7] Lancet, Oct. 15, 1904. [8] Med. Chron., July, 1904. [9] Med. Rec., Aug. 6, 1904.

